# Monkeys Monitor Human Goals in a Nonmatch-to-Goal Interactive Task

**DOI:** 10.1371/journal.pone.0032209

**Published:** 2012-02-23

**Authors:** Rossella Falcone, Emiliano Brunamonti, Stefano Ferraina, Aldo Genovesio

**Affiliations:** Department of Physiology and Pharmacology, Sapienza University of Rome, Rome, Italy; Cajal Institute, Consejo Superior de Investigaciones Científicas, Spain

## Abstract

We designed a new task, called nonmatch-to-goal, to study the ability of macaque monkeys to interact with humans in a rule-guided paradigm. In this task the monkeys were required to choose one of two targets, from a list of three. For each choice, they were required to switch from their choice on the previous trial to a different one. In a subset of trials the monkeys observed a human partner performing the task. When the human concluded his turn, the monkeys were required to switch to a new goal discarding the human's previous goal. We found that monkeys were very skillful in monitoring goals, not only of their own choice by also those of their human partner. They showed also a surprising ability to coordinate their actions, taking turns with the human partner, starting and stopping their own turn following the decision of the human partner in the task.

## Introduction

Very few studies have examined the ability of monkeys to interact with conspecifics or humans in a controlled situation. One influential line of research has shown that while observing human actions, monkeys display a shared neural representation of the action performed [1]–[3]. However, it is still debated whether these representations demonstrate that monkeys can fully understand human or conspecific actions [4]. Others studies have examined the ability of monkeys to interact by coordinating [5], [6] or cooperating [7] with each other, and their sensitivity to the reward received by other monkeys [8]. In chimpanzees, Hiraka and Fuwa [9] showed not only cooperative behavior, but also the ability to select the most efficient partner. Very little work has been done to investigate the ability of monkeys to monitor others' goals in a controlled experimental set up [6].

We wanted to test whether monkeys were able to use the active monitoring of human goals to make their own future choices. In a test of observational learning, Meunier et al. [10] showed that monkeys succeeded in learning by observing a monkey model solving discrimination learning problems, but failed with a human model. Therefore, it remains controversial whether monkeys can have the kinds of interaction with humans that they have with conspecifics.

We wanted to test whether monkeys interact successfully with humans in a more controlled experimental setting. We measured the ability of monkeys to guide their choices based on a human's previous choices. In line with our previous studies [11]–[14], we designed a nonmatch-to-goal task (NMTG) that required monkeys to switch either from their immediate previous goal or from the human partner' s goal to a new goal, in order to receive a reward.

We found that macaques were able to alternate roles with a human partner. They stopped their turns when the human intervened and promptly restarted when the human partner moved the arm away from the testing apparatus. The monkeys did not need any cue signal to instruct them that it was their turn. Instead, the monkeys monitored and adapted to the intervention of the human partner, stopping and restarting their turn accordingly. The monkeys showed high levels of performance in switching to a new goal after the human partner's choice, which was comparable to that observed for switching their own goals. This study extends to human–monkey (H–M) pairs the opportunity to investigate social cognition in a controlled laboratory setup, opening a door to future behavioral, neuropsychological, and neurophysiological studies adopting similar paradigms.

## Materials and Methods

### Animals

Animal care, housing, and experimental procedures were in conformity with the European (Directive 86/609/ECC) and Italian (D.L. 116/92) laws on the use of nonhuman primates in scientific research. The research protocol was approved by the Italian Health Ministry (Central Direction for the Veterinary Service, approval n. 199/2009-B). The housing conditions and the experimental procedures were in accordance with the European law on humane care and use of laboratory animals and complied with the recommendations of the Weatherall report (The use of non-human primates in research).

Both monkeys were monitored daily by the researchers and the animal care staff, and every second day from the veterinarian, to check the conditions of health and welfare.

To ameliorate their condition of life we routinely introduced in the home cage environment toys (often containing items of food that they liked) to promote their exploratory behaviour. At the end of each experimental session, the researcher that tested the animals spent half an hour interacting with the monkeys directly, giving for example new objects to manipulate. We think that this interaction with humans, in addition to the interaction that was part of the task performed, can help to reduce potential stress related to the experiment. Recently, to increase the level of enrichment in the animal facility room, we have also showed movies in a monitor and we are currently starting to evaluate the benefits.

### Surgical techniques

In monkey 1 the experiments were carried out while the monkey' s head was fixed. For this purpose, a head-holder was implanted. The animal was preanesthetized with ketamine (10 mg/kg, i.m.) and anesthetized with isofluoran (Abbott Laboratories) through a constant flux of isofluoran/air mixture (1–3%, to effect). Antibiotics and analgesics were administered postoperatively.

### Behavioral testing

The monkeys sat in a primate chair, monkey 1 with the head fixed and monkey 2 with the head free, facing a video touch screen (Microtouch, 19 inches, 800×600 pixel resolution) 40 cm away. For both monkeys, the human partner was standing upright, close to the animal, on the monkey's right side. For this experiment, the human partner was always the same researcher with whom the monkeys were accustomed.

**Figure 1:**
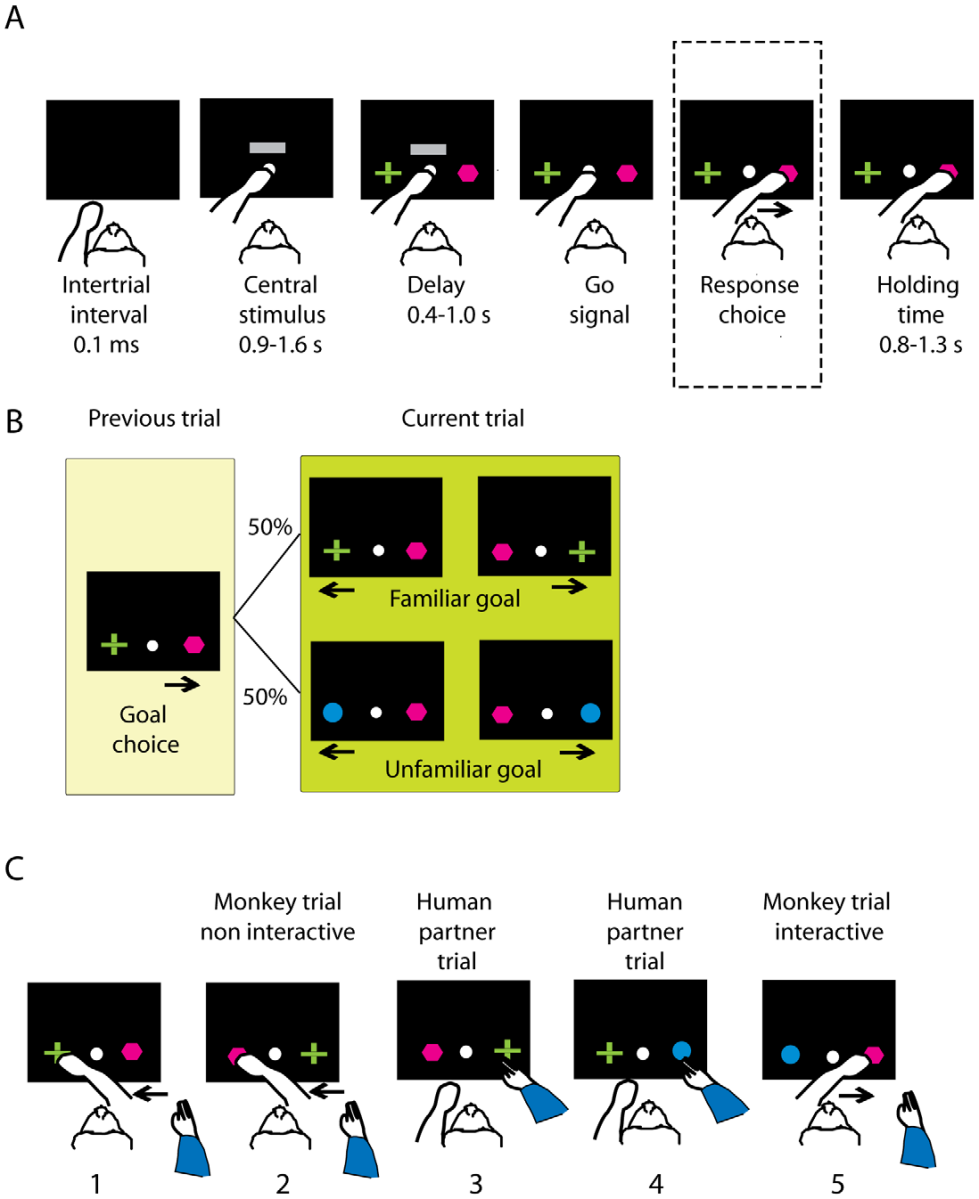
Experimental design. **A**. Sequence of task events in a trial. Each black rectangle represents the video screen. The white circle illustrates the central stimulus, the grey horizontal bar is the go cue. The green cross and the purple polygon are the two potential response goals. In this example trial, that could represent the first trial of a session, the response decision (highlighted by the braked rectangle) is toward the purple polygon. We used three potential goals in a session: a purple polygon, a green cross and a blue circle **B.** In this example sequence of trials, the previous goal was the purple polygon (left yellow box, as in the trial in A). The same goal was, by task design, presented again in the current trial (right green box), together with another potential future goal, which was either: 1) the *familiar goal* that was the goal discarded in the previous trial (green cross); or 2) the *unfamiliar* goal that was not presented in the previous trial (blue circle). **C.** Example sequence of trials with the human partner interacting with the monkey. Numbers indicate the trial position after the trial in A. Each panel represents the response choice. The correct goal (response) was always the goal that differed from the previous goal acquired either by the human agent in the *interactive* condition or by the monkey in the *noninteractive* condition. In the human trials the monkeys were required only to monitor the human partner choices. Notice that in this example sequence monkeys in trial number 5 could not choose the purple polygon based on what was their own previous choice (the purple polygon in trial 2), they had instead to choose a goal based on what the human partner chose in trial 4, that was the blue circle.

Figure 1A shows the sequence of events in the NMTG task. The trial began when a white circle, the central stimulus, appeared at the center of the video screen (Fig. 1A).

The monkeys had to touch the circle within 1 s, otherwise the trial was aborted and a new trial started. After the monkeys touched the central stimulus, a horizontal grey bar appeared. The monkeys were required to continue touching the central stimulus for 0.9–1.6 s. Next, two targets (called object goals) appeared, one to the right and one to the left of the central stimulus, after which the monkeys maintained their right hand on the central stimulus for a delay period of 0.4–1 s. The disappearance of the horizontal bar served as a go signal. The monkeys then made a reaching movement to one of the two goals, within a 3.5 s limit, and maintained the hand on the chosen goal for a hold time of 0.8–1.3 s. For correctly performed trials, three drops of fluid reward were delivered. After both correct and error trials, the two goals and the central stimulus disappeared and a 0.1 s intertrial interval with a black screen began. We randomized all the durations of the epochs in 50 ms steps. The human partner could decide to begin a ‘human trial sequence’ (1, 2, 3, or 4 trials in a row) at any time during the session. The sequence of events for these trials was identical to that of trials performed by the animals; however, reward was delivered to the monkey in all cases.

After we concluded the first experiment, to test whether the monkeys' ability to follow the human partner's trials was strictly dependent on the reward delivery, we studied the monkeys' performance in a second experiment. We introduced a visual feedback at the end of each NMTG trial, to indicate correct and error responses. Two visual stimuli, an empty square or a circle around the chosen target, were used for correct and error trials, respectively. We presented the feedback 0.5 s after the monkeys/subject touched the target for 0.8–1.3 s, followed by the reward in the case of correct trials. All the other events were the same as in the standard NMTG task. We omitted reward in a subset of correctly (one out of six) performed human trials and measured monkeys' performance in these *unrewarded interactive* trials.

### Trial types

Monkeys performed two classes of trials, classified based on who performed the trial immediately before the current trial. If the monkey performed the previous trial it was called a ‘*noninteractive* trial’, whereas if the human partner performed the previous trial it was called an ‘*interactive* trial’. Between two *interactive* trials, the monkeys performed a variable number of trials, typically three or more.

For the first trial of a session, any chosen goal was accepted as correct and rewarded. This goal was then designated as the previous goal and this designation was used for the following trial. On each trial, the previous goal was presented on the screen together with another goal. In order to receive a reward, the monkeys had to reject the previous goal and choose the alternative (future goal). Figure 1B shows the 2×2 combinations of goals and positions for the current trial when the previous goal was a purple polygon. Goal positions were always assigned pseudorandomly unless the trial followed a mistake. Two types of trials were pseudorandomly interspersed, and they differed in terms of whether the correct goal on the current trial had appeared on the previous trial. When it had, we called this trial type a ‘familiar-goal’ trial, but when the correct future goal had not appeared on the previous trials, we called it an ‘unfamiliar-goal’ trial. The familiarity is defined only with respect to the previous goal; overall, familiar and unfamiliar future goals were presented with equal probability.

If the monkeys made a mistake, by erroneously choosing the previous goal, they did not receive a reward and a correction trial followed. Correction trials consisted of a repetition of the same two goals in the same positions. The correction trial required the monkeys to choose the goal that should have been chosen during the previous trial.

In the *interactive* trials the monkeys interacted with a human partner. When the human partner moved his arm toward the screen, the monkeys drew back their arm letting the human partner perform the trial. The human partner decided the number of trials to perform: one, two, three or four (Figure 1C). When the human partner drew back his arm at the end of a trial, the monkeys again approached the central stimulus, and in this way began their next trial. In the *interactive* trials the monkeys had to monitor the human partner's choice, and later reject human's previous goal choice in favor of the alternative goal, neglecting their own previous choice.

### Data collection

A noncommercial software package, CORTEX (www.cortex.salk.edu), was used to control stimuli presentation and reward delivery and to record touches on the screen. A video camera recorded who performed each trial, the monkey or the human partner. An experimenter classified each trial offline, using the video recordings.

## Results

We studied the performance of Monkey 1 in 9 sessions and of Monkey 2 in 5 sessions. Each session was run on different days. We examined the performance of both monkeys in the two conditions: *interactive* and *noninteractive*. We found that both monkeys performed the task at a high level of performance in both conditions.

**Figure 2:**
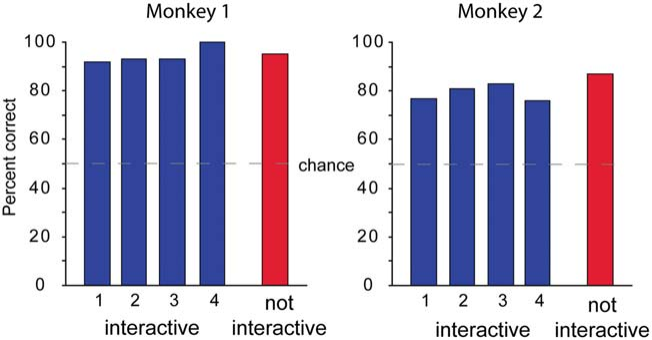
Behavioral performance. Bars show percent correct response of monkey 1 and monkey 2 for *interactive* (blue bars) and *noninteractive* trials (red bars). Numbers 1–4 indicate in the *interactive* trials the number of previous trials performed in sequence by the human partner.

Figure 2 shows the results for the *interactive* and *noninteractive* trials.

Average correct performances in the *interactive* and *noninteractive* trials were, 94% (136/145) and 95% (379/401), for monkey 1 and 79% (142/179) and 87% (321/368) for monkey 2, respectively, which was above chance level for both monkeys and both conditions (binomial test, p<0.0001). These results suggest that monkeys can recall the most recent goal chosen by their human partner, and apply this knowledge to make their own decisions in the *interactive* trials. However, the results from the *interactive* trials may also be due to the monkeys' ability to remember its own previous choice and applying one of the following rules: (1) choose own previous goal, (2) choose opposite of own previous goal. Each of these rules increases the probability of choosing the correct goal to above chance. However, the first rule works only if an odd number of trials are performed by the human partner, while the second works only after an even number of trials performed by the human partner. To reduce the possibility that the monkeys learned one of these rules, the human partner varied his number (from 1 to 4) of trials.

To quantitatively assess whether the monkeys tended to follow one of the rules previously described, we divided the errors based on the number of trials performed by the human agent before an *interactive* trial. We found that both monkeys had a comparable performance in this regard. Monkey 1 performed at 92% (61/66), 93% (42/45), 93% (13/14) and 100% (20/20), respectively after sequences of 1, 2, 3, and 4 trials of the human partner, with no significant difference (p>0.05, Chi Square test). Similarly, monkey 2 performed at 77% (53/69), 81% (44/54), 83%, (29/35), and 76% (16/21), respectively, with no significant difference between sequences (p>0.05, Chi Square test).

A similar success rate for 1 and 3 intervening human trials versus 2 and 4 of such trials shows that monkeys based their choice on what their partner did rather than the previous choices that the monkeys had made. The results for 3 and 4 intervening trials performed by the human partner show that monkeys were able to maintain a prolonged attention on the behavior of the human partner. Using the video recordings, we also examined the ability of monkeys to take turns and coordinate with the human agent. We found that both monkeys initiated trials immediately after seeing the human agent withdraw the hand from the screen, with a success of 100% in both monkeys.

We studied the performance of the two monkeys in the NMTG tasks with visual feedbacks to test whether they could perform *interactive* trials without reward delivery after correctly performed human trials (four sessions with monkey 1 and three sessions with monkey 2). We found that the average correct performance in the *interactive* trials that followed a correct but not rewarded trial were respectively, 88% (71/81) for monkey 1, and 86% (95/111) for monkey 2, and this was above chance level for both monkeys (binomial test, p<0.0001).

## Discussion

We tested two monkeys in a NMTG task, which required monkeys to interact with a human agent. Both monkeys showed an excellent performance with a very high success rate in both the *noninteractive* condition, after a self-acquired goal, and in the *interactive* condition, after the human partner-acquired goal. The second experiment showed that monkeys retained a high success rate of *interactive* trials when the reward delivery was replaced by visual feedback in human trials.

We emphasize that our study is not one of social cooperation or social learning. Nevertheless, it has several implications for future neurophysiological and behavioral studies directed to studying the cognitive processes underlying such functions. To date, only a handful of studies have shown that monkeys can learn from other monkeys [10], [15]–[18] as humans do so readily [19]. Other studies have shown that monkeys can learn the ordinal position of pictures watching other monkeys [16], [18] and monitoring their behavior [6].

It is clear that we are only beginning to understand the potentials of using monkeys as a model for studying the cognitive and affective processes underlying social behavior at the neural level [5], [6]. In this regard, it is important to mention the pioneering study of Yoshida et al. [6]. They have shown that medial prefrontal neurons are able to distinguish self from others by discharging specifically for another agent's actions, but only in a social contest. Here we extend this line of investigation, showing that monkeys can monitor the behavior of their human partner for multiple trials, alternating the role of actor and observer with a human agent.

We emphasize that, although several previous studies have shown monkeys responding to one another's behavior, there were strong doubts as to whether they could do so when interacting with humans. Questions about the ability of monkeys to interact socially with humans occurred even in behaviors much simpler than the current one, such as following the gaze of others. While it has been shown that monkeys are able to follow the gaze of a conspecific [20], [21], there has been more controversy on the ability of monkey to use the communicative gestures of humans, with some studies failing to find signs of this ability [22], [23] while others showing some evidence of this ability in macaques [24], [25]. Social learning in the form of imitating tool use by human agents has also generated controversy, with some studies finding some evidence in support of such imitation [26], [27] and others failing to find such evidence [28], [29], depending on the experimental design. When we go beyond gaze following or learning about tool use, to consider the ability of monkeys to monitor or learn more complex behaviors, previous studies have casted serious doubts on the capacity to learn from observing human behavior [17], [10]. Meunier and colleagues [10] have shown that monkeys could learn, to a certain degree, which object was associated to a reward by observing another monkey, but not a human, performing the task. That result leads to the possibility that there could be a limit to the ability of monkeys to interact with individuals of another species. Here, we showed strong evidence that, at least in the context of our laboratory task, monkeys can engage in such interactions and do so extraordinary well.

The differences between those previous results and ours might be explained by several differences in the experiment design. First, the imitative nature of the task used by Meunier et al. [10], is different from ours in that it might have required social identification. We think, however, that the main difference is the seminatural settings adopted by Meunier et al. [10], which did not require monkeys to pay attention to the other monkeys or to the human agents. In contrast, our laboratory setting posed greater demands on attention to the performance of the human model. Moreover, we showed that the human's goals could be similarly monitored both by the monkey with the head free and by the monkey with the head fixed.

We also showed that monkeys were able to wait for their turn depending on the decisions of the experimenter: a special kind of turn taking behavior. Turn-taking develops early in human infants and develops furthermore later [30], [31], and it is based at least on the abilities to distinguish self from others and to monitor others' goals and their accomplishment.

We believe that our results have relevance beyond behavioral research. Our results point to the possibility of using H–M paradigms in other fields of neuroscience. The H–M paradigm can represent a complementary approach to the monkey–monkey (M–M) paradigm that has gained prominence recently, offering new possibilities for controlling the parameters of the interaction. This could be done, for example, by using the human partner as an independent variable that can be manipulated in the study in a way that is much easier than manipulating a monkey partner. For example, it should be possible to change more flexibly the type of trials in which the interaction takes place and to predict the partner' s success rate by having different agents performing at a different level of accuracy. Nevertheless, M–M paradigm can be more adapted to study aspects such as the vicarious reinforcement [32] or the influence of social hierarchy on social interactions but new experimental designs should be developed and tested within the paradigm to investigate these topics.

Our H–M paradigm, especially if adapted to a spatial version, could be also used to study “mirror proprieties” of neurons [2], [33]–[35] with the advantage of having behavioral measures, on a trial by trial basis, to test whether the monkeys paid attention to the human actions and understood the goal and the outcome associated to the human agent's choices. In fact, in our task a failure in monitoring the actions of the human partner directly affects success on the next trial. Future studies will need to address the full range of opportunities that H–M paradigms can offer for the study of social cognition [36]. For example, we still need to address whether monkeys are able to learn from the observation of human agents. This might be done by setting the errors of the human partner as the independent variable, which would allow us to investigate learning aspects such as the effects of human partner errors on learning by observation.
